# Crystal structure of *N*′′-(2-eth­oxy-2-oxoeth­yl)-*N*,*N*,*N*′,*N*′-tetra­methyl-*N*′′-[3-(1,3,3-tri­methyl­ureido)prop­yl]guanidinium tetra­phenyl­borate

**DOI:** 10.1107/S2056989015023142

**Published:** 2015-12-09

**Authors:** Ioannis Tiritiris, Willi Kantlehner

**Affiliations:** a Fakultät Chemie/Organische Chemie, Hochschule Aalen, Beethovenstrasse 1, D-73430 Aalen, Germany

**Keywords:** crystal structure, ureido­alkyl­guanidinium, tetra­phenyl­borate, salt, C—H⋯O contacts, C—H⋯π inter­actions

## Abstract

In the title salt, C_16_H_34_N_5_O_3_^+^·C_24_H_20_B^−^, the C—N bond lengths in the cation are 1.3368 (16), 1.3375 (18) and 1.3594 (17) Å, indicating partial double-bond character. The central C atom is bonded to the three N atoms in a nearly ideal trigonal–planar geometry and the positive charge is delocal­ized in the CN_3_ plane. In the crystal, weak C—H⋯O contacts are observed between neighbouring guanidinium ions and between guanidinium ions and tetra­phenyl­borate anions. In addition, C—H⋯π inter­actions involving guanidinium H atoms and aromatic rings of the anion are present. The phenyl rings form aromatic pockets, in which the cations are embedded. This leads to the formation of a two-dimensional supramolecular pattern along the *ab* plane.

## Related literature   

For the crystal structure of *N*,*N*,*N*′,*N*′-tetra­methyl­urea, see: Frampton & Parkes (1996 ▸). For the crystal structure of *N*,*N*,*N*′,*N*′-tetra­methyl­chloro­formamidinium chloride, see: Tiritiris & Kantlehner (2008*a* ▸). For the crystal structures of alkali metal tetra­phenyl­borates, see: Behrens *et al.* (2012 ▸). For the crystal structure of 2-di­methyl­amino-1-(2-eth­oxy-2-oxoeth­yl)-3-methyl-3,4,5,6-tetra­hydro­pyrimidin-1-ium tetra­phenyl­borate, see: Tiritiris & Kantlehner (2012*a* ▸). For the crystal structure of *N*,*N*,*N*′,*N*′,*N*′′-penta­methyl-*N*′′-[3-(1,3,3-tri­methyl­ureido)prop­yl]guanidinium tetra­phenyl­borate, see: Tiritiris & Kantlehner (2012*b* ▸). For the synthesis of *N*′′-[3-(1,3,3-tri­methyl­ureido)prop­yl]-*N*,*N*,*N*′,*N*′-tetra­methyl­guanidine, see: Tiritiris & Kantlehner (2013 ▸).
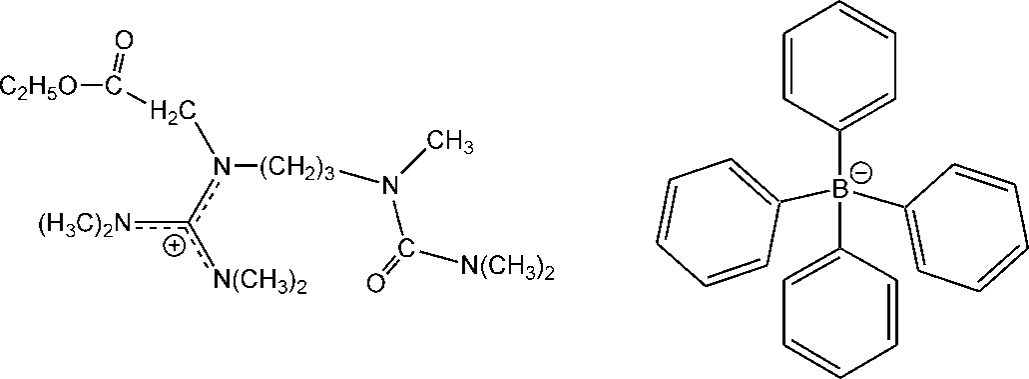


## Experimental   

### Crystal data  

C_16_H_34_N_5_O_3_^+^·C_24_H_20_B^−^
*M* *_r_* = 663.69
Monoclinic, 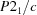

*a* = 9.6650 (3) Å
*b* = 33.8756 (9) Å
*c* = 11.1543 (5) Å
β = 93.759 (1)°
*V* = 3644.2 (2) Å^3^
*Z* = 4
Mo *K*α radiation
μ = 0.08 mm^−1^
*T* = 100 K
0.45 × 0.30 × 0.15 mm

### Data collection  

Bruker–Nonius KappaCCD diffractometer
16724 measured reflections
8666 independent reflections
6067 reflections with *I* > 2σ(*I*)
*R*_int_ = 0.048

### Refinement  

*R*[*F* ^2^ > 2σ(*F* ^2^)] = 0.048
*wR*(*F* ^2^) = 0.122
*S* = 1.06
8666 reflections
450 parameters
H-atom parameters constrained
Δρ_max_ = 0.27 e Å^−3^
Δρ_min_ = −0.31 e Å^−3^

### 

Data collection: *COLLECT* (Hooft, 2004 ▸); cell refinement: *DENZO-SMN* (Otwinowski & Minor, 1997 ▸); data reduction: *DENZO-SMN*; program(s) used to solve structure: *SHELXS97* (Sheldrick, 2008 ▸); program(s) used to refine structure: *SHELXL2014* (Sheldrick, 2015 ▸); molecular graphics: *DIAMOND* (Brandenburg & Putz, 2005 ▸); software used to prepare material for publication: *SHELXL2014*.

## Supplementary Material

Crystal structure: contains datablock(s) I, global. DOI: 10.1107/S2056989015023142/rz5179sup1.cif

Structure factors: contains datablock(s) I. DOI: 10.1107/S2056989015023142/rz5179Isup2.hkl

**Fig. 1.**
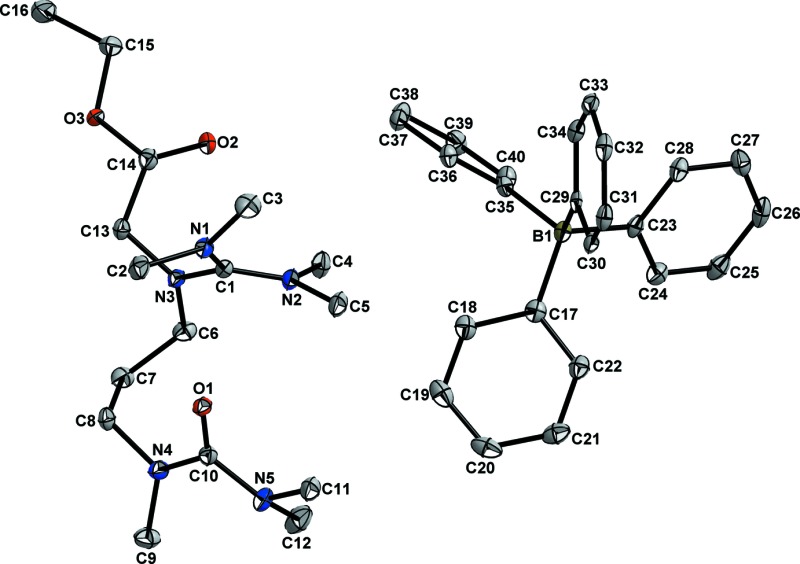
The structure of the title compound with displacement ellipsoids at the 50% probability level. All hydrogen atoms were omitted for the sake of clarity.

. DOI: 10.1107/S2056989015023142/rz5179fig1.tif
The structure of the title compound with displacement ellipsoids at the 50% probability level. All hydrogen atoms were omitted for the sake of clarity.

**Fig. 2.**
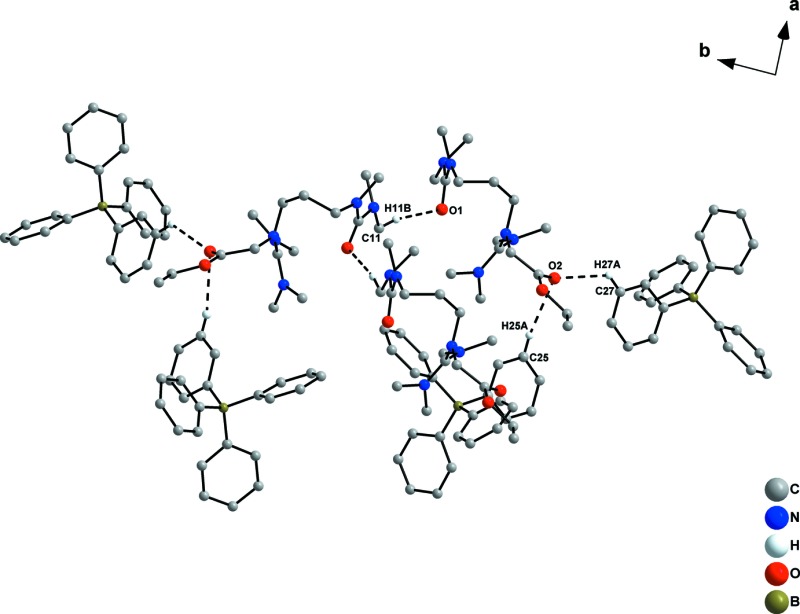
C—H···O contacts (black dashed lines) between the hydrogen atoms of tetraphenylborate ions and the oxygen atom of the cations and between the hydrogen atoms and oxygen atoms of adjacent guanidinium ions.

. DOI: 10.1107/S2056989015023142/rz5179fig2.tif
C—H⋯O contacts (black dashed lines) between the hydrogen atoms of tetra­phenyl­borate ions and the oxygen atom of the cations and between the hydrogen atoms and oxygen atoms of adjacent guanidinium ions.

**Fig. 3.**
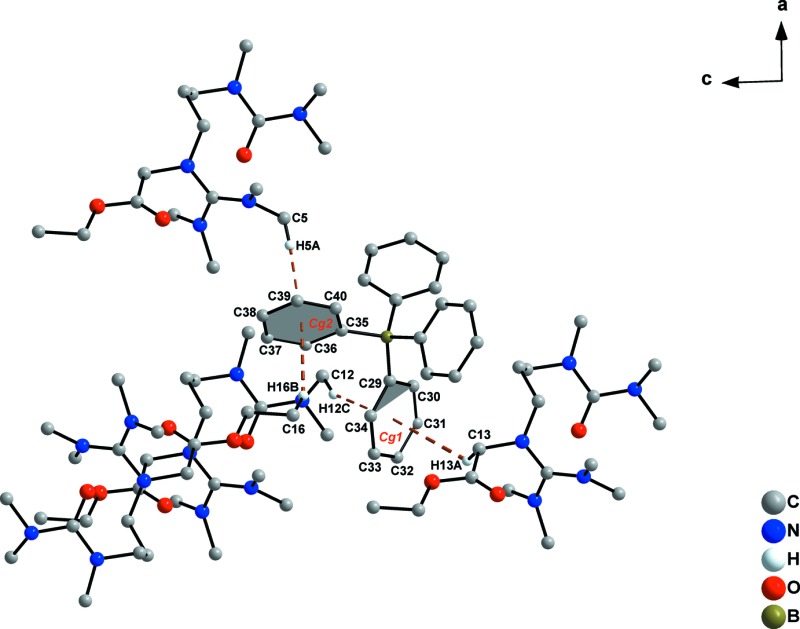
C—H···π interactions (brown dashed lines) between the hydrogen atoms of the guanidinium ion and the phenyl carbon atoms (centroids) of the tetraphenylborate ion.

. DOI: 10.1107/S2056989015023142/rz5179fig3.tif
C—H⋯π inter­actions (brown dashed lines) between the hydrogen atoms of the guanidinium ion and the phenyl carbon atoms (centroids) of the tetra­phenyl­borate ion.

CCDC reference: 1439925

Additional supporting information:  crystallographic information; 3D view; checkCIF report

## Figures and Tables

**Table 1:** Hydrogen-bond geometry (Å, °) *Cg*1 and *Cg*2 are the centroids of the C29–C34 and C35–C40 rings, respectively.

*D*—H⋯*A*	*D*—H	H⋯*A*	*D*⋯*A*	*D*—H⋯*A*
C11—H11*B*⋯O1^i^	0.98	2.73	3.687 (2)	165
C25—H25*A*⋯O2^ii^	0.95	2.72	3.617 (2)	158
C27—H27*A*⋯O2^iii^	0.95	2.71	3.392 (2)	129
C12—H12*C*⋯*Cg*1	0.98	2.64	3.541 (2)	152
C13—H13*A*⋯*Cg*1	0.99	2.91	3.432 (2)	114
C5—H5*A*⋯*Cg*2	0.98	2.89	3.868 (2)	174
C16—H16*B*⋯*Cg*2	0.98	2.66	3.542 (2)	151

Symmetry codes: (i) 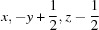; (ii) 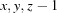; (iii) 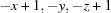.

## supplementary crystallographic information

### S1. Comment

By reaction of *N*,*N*,*N*',*N*'-tetramethylchloroformamidinium chloride (Tiritiris & Kantlehner, 2008*a*) with *N*-methyl-propane-1,3-diamine, a mixture consisting of two guanidinium chlorides and one bisguanidinium dichloride have been obtained. After treating the salt mixture with an aqueous sodium hydroxide solution, the guanidine-urea derivative *N*''-[3-(1,3,3-trimethylureido)propyl]- *N*,*N*,*N*',*N*'-tetramethylguanidine emerges as byproduct, due to partial hydrolysis of the bisguanidinium dichloride (Tiritiris & Kantlehner, 2013). As usual in guanidines, also in ureidoalkyl-guanidines electrophiles can attack on the imine nitrogen atom because it is the most basic site, giving substituted ureidoalkyl-guanidinium salts. The here presented title salt is the second one in our serie, which has been structurally characterized after anion exchange with sodium tetraphenylborate. The crystal structure analysis reveals, that the bond lengths and angles in the cation are in very good agreement with the data obtained from the structure analysis of *N*,*N*,*N*',*N*',*N*''-pentamethyl- *N*''-[3-(1,3,3-trimethylureido)propyl]guanidinium tetraphenylborate (Tiritiris & Kantlehner, 2012*b*) Prominent bond parameters in the guanidinium ion are: C1–N1 = 1.3375 (18) Å, C1–N2 = 1.3368 (16) Å and C1–N3 = 1.3594 (17) Å, indicating partial double-bond character. The N–C1–N angles are: 120.32 (12)° (N1–C1–N2), 120.64 (12)° (N2–C1–N3) and 119.04 (11)° (N1–C1–N3), which indicates a nearly ideal trigonal-planar surrounding of the carbon centre by the nitrogen atoms. The positive charge is completely delocalized on the CN_3_ plane (Fig. 1). Bond lengths in the ureido group are: C10–O1 = 1.2328 (17) Å, C10–N4 = 1.3777 (18) Å and C10–N5 = 1.3835 (17) Å. They agree very well with the data from the crystal structure analysis of solid *N*,*N*,*N*',*N*'-tetramethylurea (Frampton & Parkes, 1996). Finally, the bond lengths in the 2-ethoxy-2-oxoethyl group are comparable with the data from the structure analysis of 2-dimethylamino-1-(2-ethoxy-2-oxoethyl)-3-methyl-3,4,5,6-tetrahydropyrimidin- 1-ium tetraphenylborate (Tiritiris & Kantlehner, 2012*a*). The bond lengths and angles in the tetraphenylborate ions are in good agreement with the data from the crystal structure analysis of the alkali metal tetraphenylborates (Behrens *et al.*, 2012). C–H···O contacts between neighbouring guanidinium ions and between guanidinium ions and tetraphenylborate ions have been determined [*d*(H···O) = 2.71–2.73 Å (Tab. 1)] (Fig. 2). C–H···π interactions between the guanidinium hydrogen atoms of –N(CH_3_)_2_, –CH_2_ and –CH_3_ groups and the phenyl carbon atoms (centroids: *Cg*1 = C29–C34 and *Cg*2 = C35–C40) of the tetraphenylborate ion are also present (Fig. 3), ranging from 2.64 to 2.91 Å (Tab. 1). The phenyl rings form aromatic pockets, in which the guanidinium ions are embedded.

### S2. Experimental

The title compound was obtained by reaction of *N*''-[3-(1,3,3-trimethylureido)propyl]-*N*,*N*,*N*',*N*'-tetramethylguanidine (Tiritiris & Kantlehner, 2013) with bromoacetic acid ethyl ester in acetonitrile at room temperature. After evaporation of the solvent the crude *N*,*N*,*N*',*N*'-tetramethyl-*N*''-(2-ethoxy-2-oxoethyl)-*N*''-[3-(1,3,3-trimethylureido)propyl]guanidinium bromide (I) was washed with diethylether and dried *in vacuo*. 1.48 g (3.5 mmol) of (I) was dissolved in 20 ml acetonitrile and 1.2 g (3.5 mmol) of sodium tetraphenylborate in 20 ml acetonitrile was added. After stirring for one hour at room temperature, the precipitated sodium bromide was filtered off. The title compound crystallized from a saturated acetonitrile solution after several months at 273 K, forming colorless single crystals. Yield: 1.97 g (86%).

### S3. Refinement

The hydrogen atoms of the methyl groups were allowed to rotate with a fixed angle around the C–N and C–C bonds to best fit the experimental electron density, with *U*_iso_(H) set to 1.5 *U*_eq_(C) and d(C—H) = 0.98 Å. The remaining H atoms were placed in calculated positions with d(C—H) = 0.99 Å (H atoms in CH_2_ groups) and (C—H) = 0.95 Å (H atoms in aromatic rings). They were refined using a riding model, with *U*_iso_(H) set to 1.2*U*_eq_(C).

### Crystal data

**Table:**

C_16_H_34_N_5_O_3_^+^·C_24_H_20_B^−^	*F*(000) = 1432
*M**_r_* = 663.69	*D*_x_ = 1.210 Mg m^−^^3^
Monoclinic, *P*2_1_/*c*	Mo *K*α radiation, λ = 0.71073 Å
*a* = 9.6650 (3) Å	Cell parameters from 8389 reflections
*b* = 33.8756 (9) Å	θ = 0.4–27.9°
*c* = 11.1543 (5) Å	µ = 0.08 mm^−^^1^
β = 93.759 (1)°	*T* = 100 K
*V* = 3644.2 (2) Å^3^	Block, colorless
*Z* = 4	0.45 × 0.30 × 0.15 mm

### Data collection

**Table:**

Bruker–Nonius KappaCCD diffractometer	6067 reflections with *I* > 2σ(*I*)
Radiation source: fine-focus sealed tube	*R*_int_ = 0.048
Graphite monochromator	θ_max_ = 28.0°, θ_min_ = 1.2°
φ scans, and ω scans	*h* = −12→12
16724 measured reflections	*k* = −44→44
8666 independent reflections	*l* = −14→14

### Refinement

**Table:**

Refinement on *F*^2^	Primary atom site location: structure-invariant direct methods
Least-squares matrix: full	Secondary atom site location: difference Fourier map
*R*[*F*^2^ > 2σ(*F*^2^)] = 0.048	Hydrogen site location: inferred from neighbouring sites
*wR*(*F*^2^) = 0.122	H-atom parameters constrained
*S* = 1.06	*w* = 1/[σ^2^(*F*_o_^2^) + (0.0606*P*)^2^] where *P* = (*F*_o_^2^ + 2*F*_c_^2^)/3
8666 reflections	(Δ/σ)_max_ < 0.001
450 parameters	Δρ_max_ = 0.27 e Å^−^^3^
0 restraints	Δρ_min_ = −0.31 e Å^−^^3^

### Special details

**Table:**

Geometry. All e.s.d.'s (except the e.s.d. in the dihedral angle between two l.s. planes) are estimated using the full covariance matrix. The cell e.s.d.'s are taken into account individually in the estimation of e.s.d.'s in distances, angles and torsion angles; correlations between e.s.d.'s in cell parameters are only used when they are defined by crystal symmetry. An approximate (isotropic) treatment of cell e.s.d.'s is used for estimating e.s.d.'s involving l.s. planes.
Refinement. Refinement of *F*^2^ against ALL reflections. The weighted *R*-factor *wR* and goodness of fit *S* are based on *F*^2^, conventional *R*-factors *R* are based on *F*, with *F* set to zero for negative *F*^2^. The threshold expression of *F*^2^ > σ(*F*^2^) is used only for calculating *R*-factors(gt) *etc*. and is not relevant to the choice of reflections for refinement. *R*-factors based on *F*^2^ are statistically about twice as large as those based on *F*, and *R*- factors based on ALL data will be even larger.

### Fractional atomic coordinates and isotropic or equivalent isotropic displacement parameters (Å^2^)

**Table:**

	*x*	*y*	*z*	*U*_iso_*/*U*_eq_	
C1	0.84093 (14)	0.12384 (4)	0.92515 (11)	0.0141 (3)	
N1	0.73352 (12)	0.14132 (4)	0.97324 (9)	0.0165 (3)	
N2	0.83213 (12)	0.11315 (4)	0.80954 (10)	0.0171 (3)	
N3	0.95854 (12)	0.11695 (3)	0.99567 (9)	0.0142 (2)	
C2	0.74939 (16)	0.17100 (4)	1.06782 (12)	0.0204 (3)	
H2A	0.8478	0.1776	1.0825	0.031*	
H2B	0.7136	0.1606	1.1417	0.031*	
H2C	0.6975	0.1948	1.0428	0.031*	
C3	0.59041 (15)	0.13337 (5)	0.92991 (13)	0.0260 (4)	
H3A	0.5874	0.1097	0.8793	0.039*	
H3B	0.5539	0.1559	0.8828	0.039*	
H3C	0.5338	0.1291	0.9985	0.039*	
C4	0.89927 (16)	0.07750 (5)	0.76717 (12)	0.0225 (3)	
H4A	0.9747	0.0850	0.7171	0.034*	
H4B	0.8311	0.0615	0.7197	0.034*	
H4C	0.9369	0.0621	0.8363	0.034*	
C5	0.75132 (16)	0.13535 (5)	0.71706 (12)	0.0226 (3)	
H5A	0.6674	0.1205	0.6918	0.034*	
H5B	0.8070	0.1394	0.6478	0.034*	
H5C	0.7254	0.1610	0.7495	0.034*	
C6	1.09650 (14)	0.11774 (4)	0.94674 (12)	0.0176 (3)	
H6A	1.0870	0.1271	0.8625	0.021*	
H6B	1.1343	0.0906	0.9465	0.021*	
C7	1.19781 (15)	0.14434 (4)	1.01854 (13)	0.0204 (3)	
H7A	1.2897	0.1422	0.9848	0.024*	
H7B	1.2076	0.1346	1.1024	0.024*	
C8	1.15600 (16)	0.18780 (4)	1.01999 (12)	0.0197 (3)	
H8A	1.0612	0.1899	1.0480	0.024*	
H8B	1.2196	0.2021	1.0781	0.024*	
N4	1.15908 (12)	0.20672 (4)	0.90229 (10)	0.0188 (3)	
C9	1.28789 (15)	0.22640 (5)	0.87719 (14)	0.0235 (3)	
H9A	1.2705	0.2449	0.8104	0.035*	
H9B	1.3562	0.2067	0.8556	0.035*	
H9C	1.3237	0.2409	0.9487	0.035*	
C10	1.03676 (15)	0.21111 (4)	0.83296 (12)	0.0165 (3)	
O1	0.92302 (10)	0.20626 (3)	0.87473 (8)	0.0210 (2)	
N5	1.04850 (13)	0.22196 (4)	0.71448 (10)	0.0191 (3)	
C11	0.91878 (16)	0.23147 (5)	0.64557 (12)	0.0234 (3)	
H11A	0.8722	0.2070	0.6190	0.035*	
H11B	0.9388	0.2474	0.5754	0.035*	
H11C	0.8584	0.2464	0.6962	0.035*	
C12	1.14626 (19)	0.20145 (5)	0.64173 (14)	0.0308 (4)	
H12A	1.2216	0.1903	0.6943	0.046*	
H12B	1.1846	0.2201	0.5856	0.046*	
H12C	1.0981	0.1802	0.5965	0.046*	
C13	0.94899 (15)	0.10156 (4)	1.11681 (11)	0.0172 (3)	
H13A	0.8953	0.1203	1.1637	0.021*	
H13B	1.0434	0.0996	1.1566	0.021*	
C14	0.88068 (15)	0.06163 (4)	1.11757 (12)	0.0174 (3)	
O2	0.83182 (13)	0.04434 (4)	1.03091 (9)	0.0350 (3)	
O3	0.87943 (11)	0.04887 (3)	1.23022 (8)	0.0219 (2)	
C15	0.81131 (17)	0.01131 (5)	1.25050 (13)	0.0265 (4)	
H15A	0.8522	−0.0098	1.2028	0.032*	
H15B	0.7111	0.0131	1.2266	0.032*	
C16	0.8328 (2)	0.00251 (5)	1.38170 (14)	0.0387 (5)	
H16A	0.9322	−0.0004	1.4034	0.058*	
H16B	0.7847	−0.0220	1.3998	0.058*	
H16C	0.7957	0.0242	1.4279	0.058*	
B1	0.34042 (17)	0.11535 (5)	0.39939 (13)	0.0143 (3)	
C17	0.43843 (14)	0.15505 (4)	0.39351 (11)	0.0157 (3)	
C18	0.49782 (15)	0.17349 (4)	0.49770 (12)	0.0186 (3)	
H18A	0.4844	0.1618	0.5734	0.022*	
C19	0.57527 (15)	0.20806 (5)	0.49502 (14)	0.0223 (3)	
H19A	0.6132	0.2193	0.5680	0.027*	
C20	0.59757 (16)	0.22626 (5)	0.38682 (14)	0.0243 (3)	
H20A	0.6523	0.2495	0.3845	0.029*	
C21	0.53821 (16)	0.20976 (5)	0.28209 (13)	0.0228 (3)	
H21A	0.5500	0.2222	0.2071	0.027*	
C22	0.46146 (15)	0.17509 (4)	0.28616 (12)	0.0186 (3)	
H22A	0.4226	0.1644	0.2127	0.022*	
C23	0.35548 (15)	0.08579 (4)	0.28264 (11)	0.0151 (3)	
C24	0.47268 (15)	0.08387 (4)	0.21527 (12)	0.0195 (3)	
H24A	0.5494	0.1005	0.2370	0.023*	
C25	0.48122 (17)	0.05850 (5)	0.11770 (12)	0.0224 (3)	
H25A	0.5626	0.0583	0.0744	0.027*	
C26	0.37252 (17)	0.03359 (5)	0.08325 (12)	0.0224 (3)	
H26A	0.3771	0.0170	0.0150	0.027*	
C27	0.25708 (16)	0.03331 (5)	0.15005 (12)	0.0218 (3)	
H27A	0.1825	0.0158	0.1293	0.026*	
C28	0.25009 (15)	0.05867 (4)	0.24773 (12)	0.0178 (3)	
H28A	0.1703	0.0576	0.2930	0.021*	
C29	0.17692 (14)	0.12955 (4)	0.39947 (11)	0.0139 (3)	
C30	0.12827 (14)	0.16549 (4)	0.34958 (11)	0.0156 (3)	
H30A	0.1930	0.1828	0.3163	0.019*	
C31	−0.00979 (15)	0.17687 (4)	0.34667 (11)	0.0188 (3)	
H31A	−0.0370	0.2017	0.3130	0.023*	
C32	−0.10837 (15)	0.15240 (5)	0.39235 (11)	0.0192 (3)	
H32A	−0.2029	0.1602	0.3906	0.023*	
C33	−0.06588 (15)	0.11628 (4)	0.44077 (11)	0.0180 (3)	
H33A	−0.1319	0.0988	0.4712	0.022*	
C34	0.07363 (15)	0.10570 (4)	0.44472 (11)	0.0161 (3)	
H34A	0.1002	0.0811	0.4798	0.019*	
C35	0.38760 (14)	0.09029 (4)	0.52189 (11)	0.0154 (3)	
C36	0.33605 (15)	0.09853 (4)	0.63423 (12)	0.0176 (3)	
H36A	0.2684	0.1187	0.6392	0.021*	
C37	0.38038 (15)	0.07824 (5)	0.73862 (12)	0.0199 (3)	
H37A	0.3422	0.0846	0.8125	0.024*	
C38	0.47946 (16)	0.04899 (4)	0.73512 (12)	0.0204 (3)	
H38A	0.5105	0.0353	0.8063	0.025*	
C39	0.53299 (15)	0.03985 (4)	0.62634 (12)	0.0208 (3)	
H39A	0.6010	0.0197	0.6224	0.025*	
C40	0.48709 (15)	0.06020 (4)	0.52267 (12)	0.0185 (3)	
H40A	0.5251	0.0533	0.4490	0.022*	

### Atomic displacement parameters (Å^2^)

**Table:**

	*U*^11^	*U*^22^	*U*^33^	*U*^12^	*U*^13^	*U*^23^
C1	0.0152 (7)	0.0160 (7)	0.0112 (6)	−0.0023 (5)	0.0011 (5)	0.0007 (5)
N1	0.0116 (6)	0.0249 (7)	0.0130 (6)	−0.0006 (5)	0.0010 (5)	−0.0007 (5)
N2	0.0192 (7)	0.0210 (7)	0.0110 (6)	−0.0005 (5)	0.0000 (5)	−0.0001 (5)
N3	0.0124 (6)	0.0192 (6)	0.0111 (6)	−0.0008 (5)	0.0020 (4)	0.0011 (4)
C2	0.0204 (8)	0.0243 (8)	0.0171 (7)	0.0017 (6)	0.0058 (6)	−0.0038 (6)
C3	0.0131 (8)	0.0386 (10)	0.0260 (8)	−0.0003 (7)	0.0003 (6)	0.0040 (7)
C4	0.0281 (9)	0.0243 (8)	0.0150 (7)	0.0015 (7)	0.0009 (6)	−0.0047 (6)
C5	0.0285 (9)	0.0260 (8)	0.0127 (7)	0.0000 (7)	−0.0032 (6)	0.0018 (6)
C6	0.0125 (7)	0.0210 (8)	0.0198 (7)	0.0020 (6)	0.0040 (6)	0.0015 (6)
C7	0.0134 (8)	0.0265 (8)	0.0209 (7)	−0.0005 (6)	−0.0006 (6)	0.0057 (6)
C8	0.0190 (8)	0.0260 (8)	0.0134 (7)	−0.0039 (6)	−0.0040 (6)	0.0007 (6)
N4	0.0162 (7)	0.0230 (7)	0.0173 (6)	−0.0040 (5)	0.0010 (5)	0.0039 (5)
C9	0.0178 (8)	0.0252 (8)	0.0277 (8)	−0.0043 (6)	0.0032 (6)	0.0016 (6)
C10	0.0219 (8)	0.0147 (7)	0.0131 (7)	−0.0011 (6)	0.0012 (6)	−0.0008 (5)
O1	0.0163 (6)	0.0304 (6)	0.0163 (5)	−0.0042 (4)	0.0017 (4)	0.0025 (4)
N5	0.0219 (7)	0.0228 (7)	0.0129 (6)	0.0011 (5)	0.0030 (5)	0.0006 (5)
C11	0.0311 (9)	0.0231 (8)	0.0155 (7)	−0.0018 (7)	−0.0031 (6)	0.0019 (6)
C12	0.0411 (11)	0.0304 (9)	0.0223 (8)	0.0055 (8)	0.0122 (7)	−0.0011 (7)
C13	0.0179 (8)	0.0211 (8)	0.0123 (7)	−0.0019 (6)	−0.0019 (5)	0.0019 (6)
C14	0.0151 (7)	0.0224 (8)	0.0148 (7)	0.0004 (6)	0.0011 (5)	0.0002 (6)
O2	0.0549 (8)	0.0339 (7)	0.0159 (6)	−0.0208 (6)	0.0000 (5)	−0.0034 (5)
O3	0.0272 (6)	0.0231 (6)	0.0151 (5)	−0.0091 (5)	−0.0020 (4)	0.0049 (4)
C15	0.0315 (10)	0.0240 (8)	0.0233 (8)	−0.0118 (7)	−0.0035 (7)	0.0055 (7)
C16	0.0487 (12)	0.0364 (11)	0.0288 (9)	−0.0199 (9)	−0.0134 (8)	0.0144 (8)
B1	0.0144 (8)	0.0170 (8)	0.0113 (7)	0.0017 (6)	−0.0002 (6)	−0.0007 (6)
C17	0.0120 (7)	0.0178 (7)	0.0173 (7)	0.0045 (6)	0.0008 (5)	−0.0002 (6)
C18	0.0147 (8)	0.0220 (8)	0.0189 (7)	0.0032 (6)	−0.0008 (6)	−0.0006 (6)
C19	0.0155 (8)	0.0230 (8)	0.0281 (8)	0.0027 (6)	−0.0016 (6)	−0.0080 (6)
C20	0.0160 (8)	0.0179 (8)	0.0398 (9)	−0.0005 (6)	0.0071 (7)	−0.0037 (7)
C21	0.0223 (9)	0.0221 (8)	0.0253 (8)	0.0028 (6)	0.0114 (6)	0.0017 (6)
C22	0.0169 (8)	0.0221 (8)	0.0168 (7)	0.0022 (6)	0.0024 (6)	−0.0011 (6)
C23	0.0178 (8)	0.0166 (7)	0.0106 (6)	0.0044 (6)	−0.0012 (5)	0.0026 (5)
C24	0.0193 (8)	0.0216 (8)	0.0179 (7)	0.0008 (6)	0.0028 (6)	0.0015 (6)
C25	0.0271 (9)	0.0234 (8)	0.0173 (7)	0.0079 (7)	0.0072 (6)	0.0015 (6)
C26	0.0330 (9)	0.0219 (8)	0.0118 (7)	0.0108 (7)	−0.0018 (6)	−0.0025 (6)
C27	0.0246 (9)	0.0209 (8)	0.0191 (7)	0.0021 (6)	−0.0055 (6)	−0.0028 (6)
C28	0.0182 (8)	0.0208 (8)	0.0143 (7)	0.0039 (6)	0.0000 (6)	0.0001 (6)
C29	0.0167 (7)	0.0182 (7)	0.0065 (6)	0.0012 (6)	−0.0001 (5)	−0.0043 (5)
C30	0.0177 (8)	0.0203 (7)	0.0085 (6)	−0.0002 (6)	−0.0005 (5)	−0.0001 (5)
C31	0.0220 (8)	0.0221 (8)	0.0117 (7)	0.0058 (6)	−0.0030 (6)	−0.0011 (6)
C32	0.0165 (8)	0.0281 (8)	0.0125 (7)	0.0056 (6)	−0.0011 (6)	−0.0056 (6)
C33	0.0180 (8)	0.0260 (8)	0.0103 (6)	−0.0019 (6)	0.0027 (5)	−0.0021 (6)
C34	0.0189 (8)	0.0191 (7)	0.0103 (6)	0.0019 (6)	0.0003 (5)	−0.0002 (5)
C35	0.0154 (7)	0.0171 (7)	0.0133 (7)	−0.0023 (6)	−0.0023 (5)	−0.0008 (5)
C36	0.0170 (8)	0.0196 (8)	0.0158 (7)	0.0005 (6)	−0.0019 (6)	−0.0008 (6)
C37	0.0207 (8)	0.0272 (8)	0.0116 (7)	−0.0045 (6)	−0.0001 (6)	−0.0002 (6)
C38	0.0222 (8)	0.0226 (8)	0.0157 (7)	−0.0031 (6)	−0.0052 (6)	0.0048 (6)
C39	0.0207 (8)	0.0182 (8)	0.0225 (8)	0.0021 (6)	−0.0050 (6)	0.0005 (6)
C40	0.0196 (8)	0.0207 (8)	0.0150 (7)	0.0018 (6)	−0.0004 (6)	−0.0014 (6)

### Geometric parameters (Å, º)

**Table:**

C1—N2	1.3368 (16)	C15—H15B	0.9900
C1—N1	1.3375 (18)	C16—H16A	0.9800
C1—N3	1.3594 (17)	C16—H16B	0.9800
N1—C2	1.4583 (17)	C16—H16C	0.9800
N1—C3	1.4599 (18)	B1—C35	1.647 (2)
N2—C5	1.4608 (18)	B1—C17	1.649 (2)
N2—C4	1.4639 (18)	B1—C29	1.652 (2)
N3—C13	1.4568 (16)	B1—C23	1.657 (2)
N3—C6	1.4737 (17)	C17—C22	1.4069 (19)
C2—H2A	0.9800	C17—C18	1.4081 (19)
C2—H2B	0.9800	C18—C19	1.391 (2)
C2—H2C	0.9800	C18—H18A	0.9500
C3—H3A	0.9800	C19—C20	1.385 (2)
C3—H3B	0.9800	C19—H19A	0.9500
C3—H3C	0.9800	C20—C21	1.385 (2)
C4—H4A	0.9800	C20—H20A	0.9500
C4—H4B	0.9800	C21—C22	1.391 (2)
C4—H4C	0.9800	C21—H21A	0.9500
C5—H5A	0.9800	C22—H22A	0.9500
C5—H5B	0.9800	C23—C24	1.401 (2)
C5—H5C	0.9800	C23—C28	1.408 (2)
C6—C7	1.520 (2)	C24—C25	1.393 (2)
C6—H6A	0.9900	C24—H24A	0.9500
C6—H6B	0.9900	C25—C26	1.382 (2)
C7—C8	1.527 (2)	C25—H25A	0.9500
C7—H7A	0.9900	C26—C27	1.382 (2)
C7—H7B	0.9900	C26—H26A	0.9500
C8—N4	1.4630 (17)	C27—C28	1.3925 (19)
C8—H8A	0.9900	C27—H27A	0.9500
C8—H8B	0.9900	C28—H28A	0.9500
N4—C10	1.3777 (18)	C29—C34	1.404 (2)
N4—C9	1.4554 (18)	C29—C30	1.4066 (19)
C9—H9A	0.9800	C30—C31	1.387 (2)
C9—H9B	0.9800	C30—H30A	0.9500
C9—H9C	0.9800	C31—C32	1.385 (2)
C10—O1	1.2328 (17)	C31—H31A	0.9500
C10—N5	1.3835 (17)	C32—C33	1.389 (2)
N5—C12	1.4610 (19)	C32—H32A	0.9500
N5—C11	1.4630 (18)	C33—C34	1.393 (2)
C11—H11A	0.9800	C33—H33A	0.9500
C11—H11B	0.9800	C34—H34A	0.9500
C11—H11C	0.9800	C35—C40	1.401 (2)
C12—H12A	0.9800	C35—C36	1.4064 (19)
C12—H12B	0.9800	C36—C37	1.3952 (19)
C12—H12C	0.9800	C36—H36A	0.9500
C13—C14	1.505 (2)	C37—C38	1.380 (2)
C13—H13A	0.9900	C37—H37A	0.9500
C13—H13B	0.9900	C38—C39	1.385 (2)
C14—O2	1.2004 (16)	C38—H38A	0.9500
C14—O3	1.3296 (16)	C39—C40	1.3936 (19)
O3—C15	1.4570 (17)	C39—H39A	0.9500
C15—C16	1.495 (2)	C40—H40A	0.9500
C15—H15A	0.9900		
			
N2—C1—N1	120.32 (12)	O3—C15—C16	106.90 (12)
N2—C1—N3	120.64 (12)	O3—C15—H15A	110.3
N1—C1—N3	119.04 (11)	C16—C15—H15A	110.3
C1—N1—C2	123.21 (12)	O3—C15—H15B	110.3
C1—N1—C3	121.94 (12)	C16—C15—H15B	110.3
C2—N1—C3	114.78 (12)	H15A—C15—H15B	108.6
C1—N2—C5	122.62 (12)	C15—C16—H16A	109.5
C1—N2—C4	122.24 (12)	C15—C16—H16B	109.5
C5—N2—C4	115.11 (11)	H16A—C16—H16B	109.5
C1—N3—C13	119.79 (11)	C15—C16—H16C	109.5
C1—N3—C6	121.67 (11)	H16A—C16—H16C	109.5
C13—N3—C6	117.64 (11)	H16B—C16—H16C	109.5
N1—C2—H2A	109.5	C35—B1—C17	108.95 (11)
N1—C2—H2B	109.5	C35—B1—C29	111.24 (11)
H2A—C2—H2B	109.5	C17—B1—C29	108.33 (11)
N1—C2—H2C	109.5	C35—B1—C23	107.87 (11)
H2A—C2—H2C	109.5	C17—B1—C23	112.42 (11)
H2B—C2—H2C	109.5	C29—B1—C23	108.05 (11)
N1—C3—H3A	109.5	C22—C17—C18	114.18 (13)
N1—C3—H3B	109.5	C22—C17—B1	123.43 (12)
H3A—C3—H3B	109.5	C18—C17—B1	122.23 (12)
N1—C3—H3C	109.5	C19—C18—C17	123.13 (13)
H3A—C3—H3C	109.5	C19—C18—H18A	118.4
H3B—C3—H3C	109.5	C17—C18—H18A	118.4
N2—C4—H4A	109.5	C20—C19—C18	120.55 (13)
N2—C4—H4B	109.5	C20—C19—H19A	119.7
H4A—C4—H4B	109.5	C18—C19—H19A	119.7
N2—C4—H4C	109.5	C19—C20—C21	118.42 (14)
H4A—C4—H4C	109.5	C19—C20—H20A	120.8
H4B—C4—H4C	109.5	C21—C20—H20A	120.8
N2—C5—H5A	109.5	C20—C21—C22	120.35 (14)
N2—C5—H5B	109.5	C20—C21—H21A	119.8
H5A—C5—H5B	109.5	C22—C21—H21A	119.8
N2—C5—H5C	109.5	C21—C22—C17	123.34 (13)
H5A—C5—H5C	109.5	C21—C22—H22A	118.3
H5B—C5—H5C	109.5	C17—C22—H22A	118.3
N3—C6—C7	112.52 (11)	C24—C23—C28	114.65 (12)
N3—C6—H6A	109.1	C24—C23—B1	124.43 (12)
C7—C6—H6A	109.1	C28—C23—B1	120.84 (12)
N3—C6—H6B	109.1	C25—C24—C23	122.61 (14)
C7—C6—H6B	109.1	C25—C24—H24A	118.7
H6A—C6—H6B	107.8	C23—C24—H24A	118.7
C6—C7—C8	114.54 (12)	C26—C25—C24	120.71 (14)
C6—C7—H7A	108.6	C26—C25—H25A	119.6
C8—C7—H7A	108.6	C24—C25—H25A	119.6
C6—C7—H7B	108.6	C27—C26—C25	118.69 (13)
C8—C7—H7B	108.6	C27—C26—H26A	120.7
H7A—C7—H7B	107.6	C25—C26—H26A	120.7
N4—C8—C7	113.07 (12)	C26—C27—C28	120.00 (14)
N4—C8—H8A	109.0	C26—C27—H27A	120.0
C7—C8—H8A	109.0	C28—C27—H27A	120.0
N4—C8—H8B	109.0	C27—C28—C23	123.21 (14)
C7—C8—H8B	109.0	C27—C28—H28A	118.4
H8A—C8—H8B	107.8	C23—C28—H28A	118.4
C10—N4—C9	123.77 (12)	C34—C29—C30	114.41 (13)
C10—N4—C8	118.94 (12)	C34—C29—B1	122.37 (12)
C9—N4—C8	116.18 (11)	C30—C29—B1	123.15 (12)
N4—C9—H9A	109.5	C31—C30—C29	123.08 (13)
N4—C9—H9B	109.5	C31—C30—H30A	118.5
H9A—C9—H9B	109.5	C29—C30—H30A	118.5
N4—C9—H9C	109.5	C32—C31—C30	120.59 (14)
H9A—C9—H9C	109.5	C32—C31—H31A	119.7
H9B—C9—H9C	109.5	C30—C31—H31A	119.7
O1—C10—N4	121.81 (12)	C31—C32—C33	118.52 (14)
O1—C10—N5	121.83 (13)	C31—C32—H32A	120.7
N4—C10—N5	116.34 (13)	C33—C32—H32A	120.7
C10—N5—C12	120.06 (12)	C32—C33—C34	119.99 (14)
C10—N5—C11	116.06 (12)	C32—C33—H33A	120.0
C12—N5—C11	112.09 (12)	C34—C33—H33A	120.0
N5—C11—H11A	109.5	C33—C34—C29	123.39 (13)
N5—C11—H11B	109.5	C33—C34—H34A	118.3
H11A—C11—H11B	109.5	C29—C34—H34A	118.3
N5—C11—H11C	109.5	C40—C35—C36	114.96 (12)
H11A—C11—H11C	109.5	C40—C35—B1	122.18 (12)
H11B—C11—H11C	109.5	C36—C35—B1	122.81 (12)
N5—C12—H12A	109.5	C37—C36—C35	122.58 (13)
N5—C12—H12B	109.5	C37—C36—H36A	118.7
H12A—C12—H12B	109.5	C35—C36—H36A	118.7
N5—C12—H12C	109.5	C38—C37—C36	120.38 (13)
H12A—C12—H12C	109.5	C38—C37—H37A	119.8
H12B—C12—H12C	109.5	C36—C37—H37A	119.8
N3—C13—C14	112.41 (11)	C37—C38—C39	119.02 (13)
N3—C13—H13A	109.1	C37—C38—H38A	120.5
C14—C13—H13A	109.1	C39—C38—H38A	120.5
N3—C13—H13B	109.1	C38—C39—C40	119.96 (14)
C14—C13—H13B	109.1	C38—C39—H39A	120.0
H13A—C13—H13B	107.9	C40—C39—H39A	120.0
O2—C14—O3	125.09 (14)	C39—C40—C35	123.10 (13)
O2—C14—C13	125.70 (13)	C39—C40—H40A	118.5
O3—C14—C13	109.19 (11)	C35—C40—H40A	118.5
C14—O3—C15	117.58 (11)		
			
N2—C1—N1—C2	−146.94 (13)	C18—C17—C22—C21	1.1 (2)
N3—C1—N1—C2	33.4 (2)	B1—C17—C22—C21	176.50 (13)
N2—C1—N1—C3	29.8 (2)	C35—B1—C23—C24	−94.23 (15)
N3—C1—N1—C3	−149.78 (13)	C17—B1—C23—C24	25.92 (18)
N1—C1—N2—C5	33.0 (2)	C29—B1—C23—C24	145.41 (13)
N3—C1—N2—C5	−147.38 (14)	C35—B1—C23—C28	82.39 (15)
N1—C1—N2—C4	−144.91 (14)	C17—B1—C23—C28	−157.47 (12)
N3—C1—N2—C4	34.7 (2)	C29—B1—C23—C28	−37.98 (16)
N2—C1—N3—C13	−136.39 (13)	C28—C23—C24—C25	3.1 (2)
N1—C1—N3—C13	43.24 (19)	B1—C23—C24—C25	179.88 (13)
N2—C1—N3—C6	32.45 (19)	C23—C24—C25—C26	−0.4 (2)
N1—C1—N3—C6	−147.92 (13)	C24—C25—C26—C27	−2.3 (2)
C1—N3—C6—C7	128.32 (14)	C25—C26—C27—C28	1.9 (2)
C13—N3—C6—C7	−62.61 (16)	C26—C27—C28—C23	1.0 (2)
N3—C6—C7—C8	−62.20 (16)	C24—C23—C28—C27	−3.4 (2)
C6—C7—C8—N4	−67.10 (16)	B1—C23—C28—C27	179.65 (12)
C7—C8—N4—C10	99.05 (15)	C35—B1—C29—C34	−37.55 (17)
C7—C8—N4—C9	−92.53 (15)	C17—B1—C29—C34	−157.27 (12)
C9—N4—C10—O1	−154.18 (14)	C23—B1—C29—C34	80.68 (15)
C8—N4—C10—O1	13.3 (2)	C35—B1—C29—C30	145.51 (12)
C9—N4—C10—N5	24.7 (2)	C17—B1—C29—C30	25.78 (16)
C8—N4—C10—N5	−167.82 (13)	C23—B1—C29—C30	−96.26 (14)
O1—C10—N5—C12	−133.66 (16)	C34—C29—C30—C31	0.94 (18)
N4—C10—N5—C12	47.47 (19)	B1—C29—C30—C31	178.11 (12)
O1—C10—N5—C11	6.4 (2)	C29—C30—C31—C32	−1.0 (2)
N4—C10—N5—C11	−172.48 (12)	C30—C31—C32—C33	−0.12 (19)
C1—N3—C13—C14	61.55 (17)	C31—C32—C33—C34	1.23 (19)
C6—N3—C13—C14	−107.74 (14)	C32—C33—C34—C29	−1.3 (2)
N3—C13—C14—O2	−3.7 (2)	C30—C29—C34—C33	0.22 (18)
N3—C13—C14—O3	178.03 (12)	B1—C29—C34—C33	−176.97 (12)
O2—C14—O3—C15	−1.2 (2)	C17—B1—C35—C40	−91.78 (15)
C13—C14—O3—C15	177.14 (12)	C29—B1—C35—C40	148.86 (13)
C14—O3—C15—C16	176.18 (14)	C23—B1—C35—C40	30.52 (18)
C35—B1—C17—C22	156.72 (13)	C17—B1—C35—C36	85.35 (16)
C29—B1—C17—C22	−82.12 (15)	C29—B1—C35—C36	−34.01 (18)
C23—B1—C17—C22	37.21 (18)	C23—B1—C35—C36	−152.35 (13)
C35—B1—C17—C18	−28.25 (17)	C40—C35—C36—C37	−0.1 (2)
C29—B1—C17—C18	92.91 (15)	B1—C35—C36—C37	−177.43 (13)
C23—B1—C17—C18	−147.76 (13)	C35—C36—C37—C38	0.6 (2)
C22—C17—C18—C19	−1.4 (2)	C36—C37—C38—C39	−0.7 (2)
B1—C17—C18—C19	−176.82 (13)	C37—C38—C39—C40	0.3 (2)
C17—C18—C19—C20	0.1 (2)	C38—C39—C40—C35	0.2 (2)
C18—C19—C20—C21	1.5 (2)	C36—C35—C40—C39	−0.3 (2)
C19—C20—C21—C22	−1.8 (2)	B1—C35—C40—C39	177.04 (13)
C20—C21—C22—C17	0.4 (2)		

### Hydrogen-bond geometry (Å, º)

*Cg*1 and *Cg*2 are the centroids of the C29–C34 and C35–C40 rings, respectively.

**Table:**

*D*—H···*A*	*D*—H	H···*A*	*D*···*A*	*D*—H···*A*
C11—H11*B*···O1^i^	0.98	2.73	3.687 (2)	165
C25—H25*A*···O2^ii^	0.95	2.72	3.617 (2)	158
C27—H27*A*···O2^iii^	0.95	2.71	3.392 (2)	129
C12—H12*C*···*Cg*1	0.98	2.64	3.541 (2)	152
C13—H13*A*···*Cg*1	0.99	2.91	3.432 (2)	114
C5—H5*A*···*Cg*2	0.98	2.89	3.868 (2)	174
C16—H16*B*···*Cg*2	0.98	2.66	3.542 (2)	151

Symmetry codes: (i) *x*, −*y*+1/2, *z*−1/2; (ii) *x*, *y*, *z*−1; (iii) −*x*+1, −*y*, −*z*+1.
