# Supplementary figures and images for: Crystal structure of N′′-(2-eth­oxy-2-oxoeth­yl)-N,N,N′,N′-tetra­methyl-N′′-[3-(1,3,3-tri­methyl­ureido)prop­yl]guanidinium tetra­phenyl­borate
Source: Acta Crystallogr E Crystallogr Commun. 2015 Dec 9;71(Pt 12):o1026–7. doi: 10.1107/S2056989015023142 (PMC4719960; doi:10.1107/S2056989015023142)

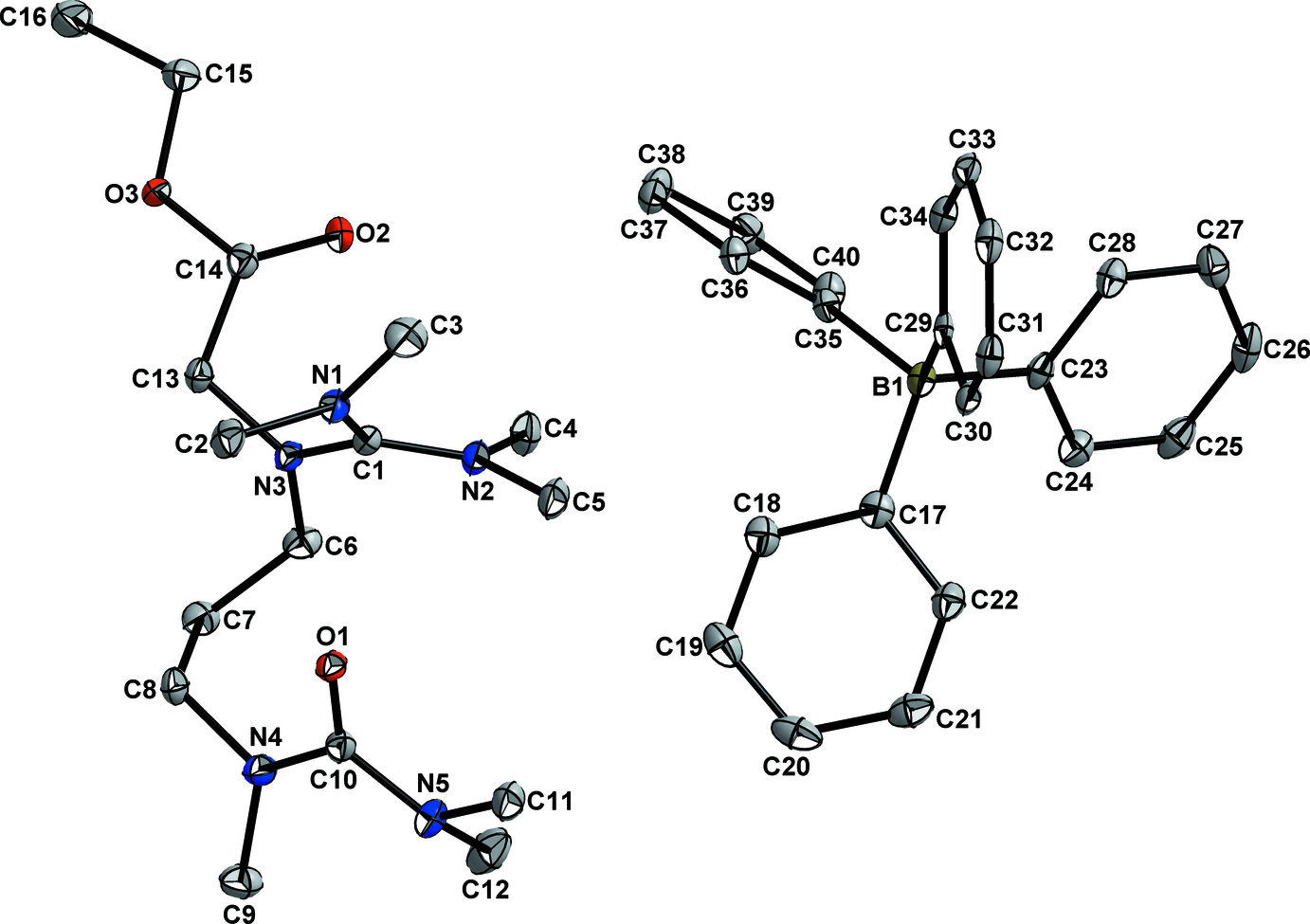

Supplement: Supplementary file 3 [file e-71-o1026-fig1.tif]

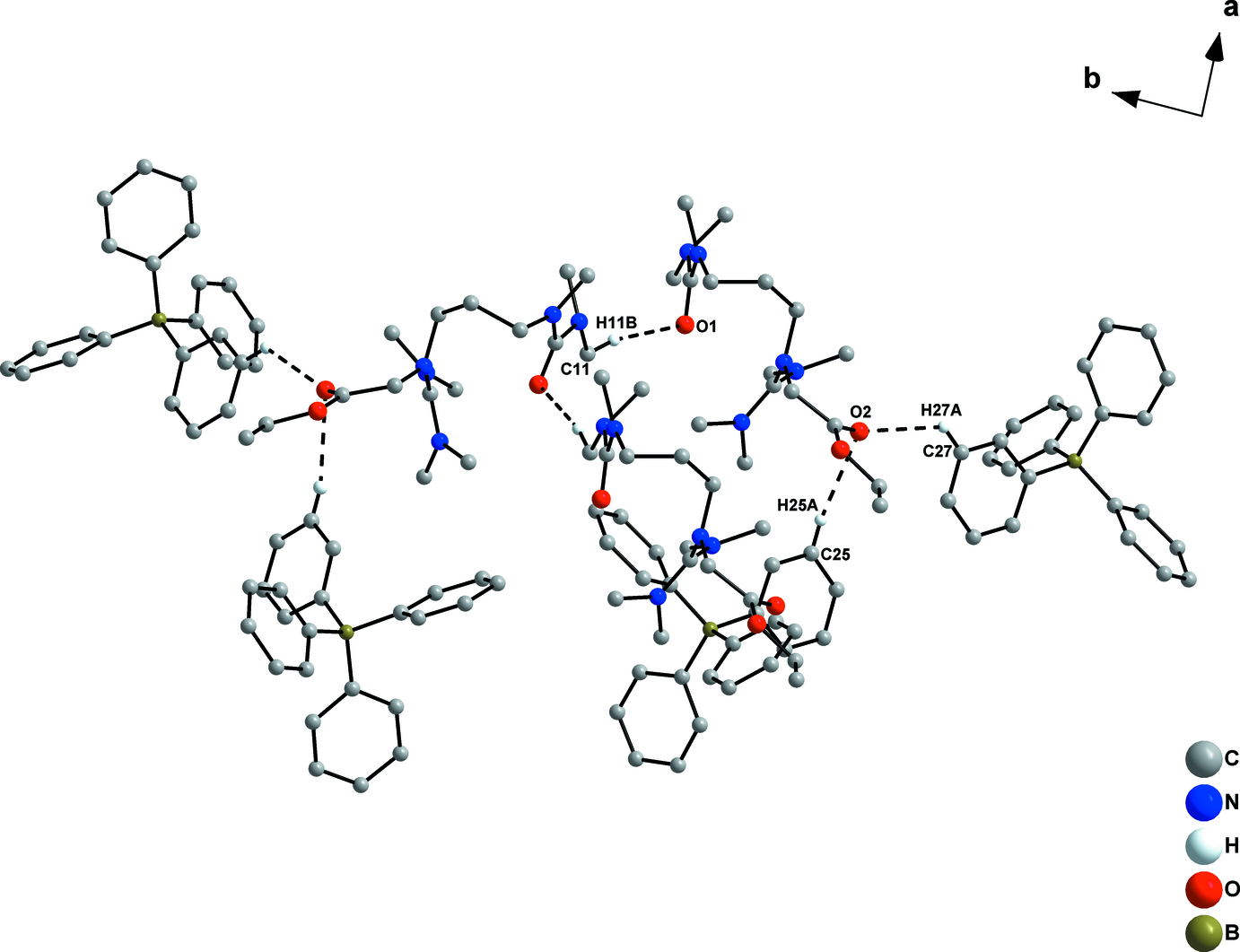

Supplement: Supplementary file 4 [file e-71-o1026-fig2.tif]

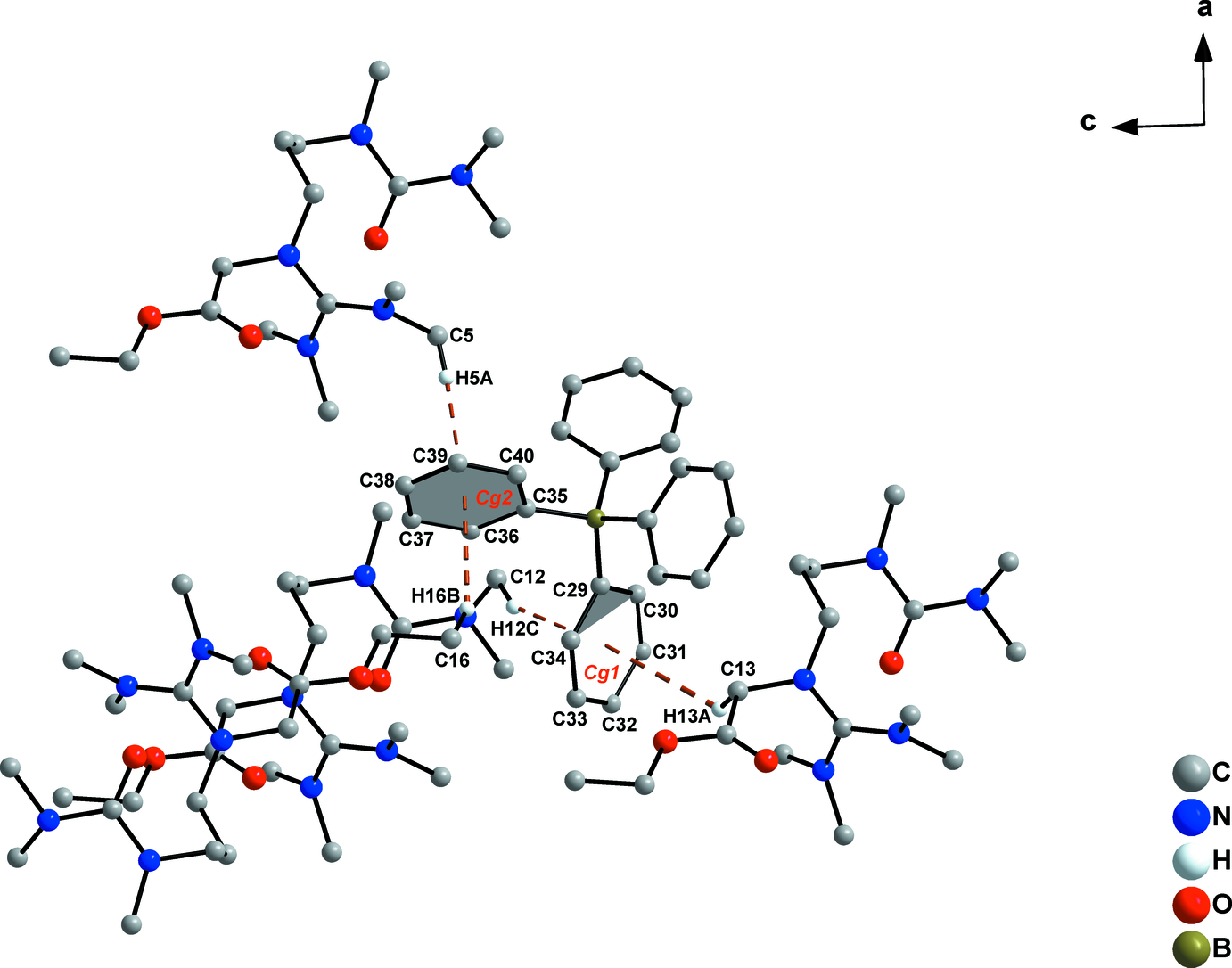

Supplement: Supplementary file 5 [file e-71-o1026-fig3.tif]
